# Factors associated with visual function among computer-based administrative workers: a Brazilian cross-sectional study

**DOI:** 10.47626/1679-4435-2022-861

**Published:** 2023-08-08

**Authors:** Eduardo Costa Sá, Maria Carmen Martinez, João Silvestre Silva-Junior, Frida Marina Fischer

**Affiliations:** 1 Departamento de Patologia da Universidade Federal de São Paulo/Escola Paulista de Medicina, São Paulo, SP, Brazil; 2 WAF Informática e Saúde Ltda., São Paulo, SP, Brazil; 3 Departamento de Medicina, Centro Universitário São Camilo, São Paulo, SP, Brazil; 4 Departamento de Saúde Ambiental, Universidade de São Paulo, São Paulo, SP, Brazil

**Keywords:** asthenopia, ergonomics, administrative personnel, occupational health, astenopia, ergonomia, pessoal administrativo, saúde do trabalhador

## Abstract

**Introduction:**

Several studies have shown that eye and vision problems are among the most significant issues reported by individuals who use computers at work.

**Objectives:**

To investigate individual and occupational environmental factors associated with visual function among workers who perform computer-based administrative tasks.

**Methods:**

This is a cross-sectional study conducted in 2014-2015 with 303 workers of a public hospital in the city of São Paulo, Brazil. The participants answered a structured questionnaire, including the 25-Item National Eye Institute Visual Function Questionnaire. Statistical analyses used descriptive analysis, tests of association and multiple linear regression analysis.

**Results:**

Most participants were female (61.1%); the mean age was 46.0 (standard deviation [SD]) ± 12.5, and approximately 91.7% of them reported wearing corrective lenses. Regarding visual function, the mean score at the 25-Item National Eye Institute Visual Function Questionnaire was 78.0, SD ±7.1. A regression analysis showed that visual function declined with age (ß −0.218; 95%CI −0.276--0.16l) and effort at work (ß −0.656; 95%CI −0.928--0.383).

**Conclusions:**

The mean quality of visual health in the studied group was good. The younger the age and the lower the effort at work, the better the visual function. Our results point to the relevance of establishing periodical and preventive health actions, including eye health assessments.

## Abstract (pt)

**Introdução:**

Vários estudos mostraram que os problemas oculares e visuais são distúrbios importantes relatados por usuários de computador.

**Objetivos:**

Investigar os fatores ambientais individuais e ocupacionais associados à função visual em trabalhadores administrativos usuários de computador.

**Métodos:**

Estudo transversal realizado em 2014-2015, com 303 trabalhadores de um hospital público da cidade de São Paulo, Brasil. Os participantes responderam a um questionário estruturado, incluindo o Questionário de Função Visual do National Eye Institute de 25 Itens. A análise estatística utilizou variáveis descritivas e qualitativas e uma análise de regressão linear múltipla.

**Resultados:**

A maioria dos participantes era do sexo feminino (61,1%); a média de idade era de 46,0 (desvio-padrão) ± 12,5, aproximadamente 91,7% deles relataram o uso de lentes corretivas. O escore médio do Questionário de Função Visual do National Eye Institute de 25 Itens foi de 78,0 desvios padrão ± 7,1. A análise de regressão mostrou que a função visual diminuiu com o aumento da idade (ß −0,218; IC95% −0,276--0,161) e esforços no trabalho (ß −0,656; IC95% −0,928--0,383).

**Conclusões:**

A qualidade média da saúde visual era boa no grupo. A função visual era melhor entre os mais jovens e com percepção de menor esforço no trabalho. Os resultados apontam para a relevância de manter ações periódicas, como a avaliação da saúde ocular.

## INTRODUCTION

Information technology has expanded in the past decades, resulting in an increased use of computers at the workplace.^1^ A survey conducted in Europe found that around 30% of workers continually use computers throughout working hours.^2^ The proper development of daily activities and work tasks depends on vision for reading and interacting with objects and people.^3^ The notion of visual function points to a broader conception of vision, understood as the set of mechanisms through which individuals interpret images and their visual environment.^4^

Eye and vision problems are among the most significant health complaints of individuals who use computers at work.^5^ Asthenopia, ie, visual fatigue, is one of the most common types of visual impairment. As a rule, the term asthenopia is used to designate any subjective symptom or discomfort related with the use of the eyes.^6^ In addition, its frequency is increasing among workers in jobs that demand high visual accuracy, such as telemarketing operators.^7,8^ A study conducted in Brazil found a prevalence of 54.6% of visual symptoms associated with computer use among telemarketing operators.^9^

Computer vision syndrome (CVS), also known as digital eye strain, is a disturbance of visual function and can be characterized as the presence of one or more symptoms derived from the use of computer screens, such as tired eyes, eyestrain, burning eyes, eye irritation, redness, blurred vision, and dry eyes.^10,11^ The global prevalence of CVS is estimated at over 70%.^5^ Studies conducted in the United States report that 90% of the 70 million workers who use computers for more than 3 hours per day exhibited some clinical sign of CVS.^10^

CVS has a multifactorial origin^12^ and its known causes are categorized as intrinsic^13^ or extrinsic, the latter being further divided into environmental and ocular causes.^10^ Among the intrinsic factors, muscle-related causes of visual fatigue prevail.^10^ The extrinsic ocular factors comprise a reduced blinking rate, increased exposure of eye surface, the use of contact lenses or medications, and the presence of systemic and/or external eye diseases.^10^

Environmental factors are related to poor workplace conditions and include lighting,^14^ dust and dryness of the air, and improper shape and position of chairs.^10^ They might demand continuous changes in visual accommodation and convergence due to the need to focus on different distances and directions, which requires adequate coordination of eye movement for accomplishing binocular vision through image fusion.^15^

A possible relationship between vision disturbance and psychosocial factors at work is based on the idea that visual disorders are related to the intensity and duration of visual demands, the workers’ self-perceived working conditions, and the pathophysiological characteristics of each individual’s visual system.^5,16^ In most cases, symptoms of visual impairment develop when the visual demands posed by the work tasks exceed the visual capacity of individuals to perform them in a comfortable manner.^1^

Considering the high prevalence of visual function impairment among workers who regularly use computers at work and the scarcity of studies about risk factors for this outcome, the aim of the present study was to investigate individual and occupational environmental factors associated with visual function among workers who perform computer-based administrative tasks.

## METHODS

### STUDY POPULATION AND DESIGN

The present cross-sectional observational study was conducted between 2014 and 2015 with administrative employees at a tertiary public hospital in São Paulo, Brazil. The population was 772 workers, but only 437 met the following inclusion criteria: performing administrative tasks, using computers for at least 4 hours a day, working daytime hours, and having worked for at least 1 year on the current position. Since 125 (28.6%) workers were excluded for being on sick (n = 119) or maternity (n = 6) leaves, 312 eligible employees remained, but nine refused participation. Therefore, 303 (97.1%) employees participated in the study.

The participants worked on workstations distributed across the six floors of the administration building. According to the institutional “Environmental Risk Prevention Program” (Programa de Prevenção de Riscos Ambientais [PPRA]), the environmental lighting system included natural and artificial sources to direct the light flux. No local or supplementary light sources were present at the individual workstations. According to the PPRA, the measured illuminance in the investigated areas varied from 480 to 500 lux.

### DATA COLLECTION AND STUDY VARIABLES

Data were collected during the ophthalmological examination performed in the periodic medical consultation, when participants filled out the self-reporting questionnaire on individual characteristics (sex, age, educational level, marital status, number of household residents, family income, routine medical examinations, smoking, alcohol intake, physical activity, duration of sleep during the workweek, clinical visual disturbances, and use of glasses or contact lenses), occupational aspects/working conditions (employment status, tenure at the current position and at the institution, weekly working hours at the hospital, daily screen time, and environmental conditions such as body postures, work tools, and psychosocial stressors) and visual function.

The third part of the questionnaire focused on aspects of the psychosocial environment at work by means of the Job Stress Scale (JSS) and the Effort-Reward Imbalance scale (ERI). The JSS is an abridged version of the Job Content Questionnaire (JCQ) based on the demand-control model and validated for use in Brazil.^17^ The ratio between the demand and control scores provided a work strain score in which higher scores represented greater strain. The transcultural adaptation of ERI for Brazilian Portuguese was also employed.^18^ The ratio between effort and reward scores provided a score for imbalance.

The outcome variable was visual function. It was assessed with the National Eye Institute Visual Function Questionnaire (NEI VFQ-25) in its Brazilian Portuguese version.^19^ NEI VFQ-25 comprises 25 questions clustered into the following 12 subdomains, with scores ranging from 0 to 100%: general health, global vision, ocular pain, difficulty with near vision activities, difficulty with distance vision activities, limitations in social functioning due to vision, mental health symptoms due to vision, role limitations due to vision, dependency on others due to vision, driving difficulties, and limitations with color and peripheral vision. The global NEI VFQ-25 score ranges from 0 to 100: the higher the score, the better the visual function.

### STATISTICAL ANALYSIS

Descriptive analysis was based on the calculation of means, medians, standard deviation (SD), and minimum and maximum values for quantitative variables, as well as proportions for qualitative variables.

The Kolmogorov-Smirnov test was used to investigate the adherence of NEI VFQ-25 scores to the normal distribution; the result, p = 0.289, enabled the use of parametric tests in the statistical analysis.

A univariate analysis of the factors associated with NEI VFQ-25 scores was performed using Pearson’s correlation coefficient for quantitative variables; an analysis of variance (ANOVA) was performed for categorical variables with constant variance, and the Mann-Whitney (dichotomous) and Kruskal-Wallis (three or more categories) tests were done for variables without constant variance. A Tukey’s post hoc test for multiple comparisons was then performed. The homogeneity of variances was assessed by Levene’s test.

A multiple forward stepwise model was fit including the variables that exhibited p < 0.20 on the univariate analysis; the p-value determined the order of inclusion into the multiple model. Qualitative variables were transformed into dummy variables, considering the category with the highest mean score on NEI VFQ-25 as reference. Potential confounding and interaction effects were tested. The descriptive level of p < 0.05 was adopted.

### ETHICAL ISSUES

The study was approved by the Research Ethics Committee of Escola de Saúde Pública, Universidade de São Paulo (USP) (ruling no. 257,510) and the Research Ethics Committee of Hospital das Clínicas, Faculdade de Medicina, USP (ruling no. 705,863) and complied with the Declaration of Helsinki. The recruited employees agreed to participate by signing an informed consent form.

## RESULTS

### DESCRIPTIVE ANALYSIS

The total population of workers comprised 437 individuals, but only 312 of them were eligible for the study. The participants were 303 (97.1%) eligible employees, and non-participants did not differ from participants as to their age range or tenure at the institution; however, there was a statistically significant difference (p = 0.013) in sex, since losses were higher among men (4.6%) compared to women (0%).

Table 1 describes the study population as to their personal characteristics. Around 61.1% of the participants were female; 34% had complete secondary education; 62% were married or had a partner; 72.9% lived in households with up to three people; and 29% reported a family income of less than 5.2 times the Brazilian minimum monthly wage. Around 66.7% of the participants reported they underwent routine medical examinations at intervals of less than 2 years; 95.7% did not smoke; 72.6% consumed alcohol one or more times per week; 58.4% had regular physical activity; 93.6% reported sleeping 6 or more hours per night during the workweek.

**Table 1:** Visual function scores of computer-based administrative workers of a public hospital according to their individual characteristics, São Paulo, 2015 (n = 303)

Variables	n	%	Mean (SD)	p-value
Sex				
Male	118	38.9	78.78 (7.04)	0.122*
Female	185	61.1	77.5 (7.04)	
Education				
Incomplete secondary education or less	103	34.0	77.6 (6.6)	0.057*
Incomplete higher education‡	60	19.8	80.2 (7.2)	
Complete higher education§	100	33.0	77.1 (7.6)	
Graduate education	38	12.5	78.1 (6.3)	
Marital status				
Single	100	33.0	80.1 (6.9)	0.001*
Married/with partner	188	62.0	77.0 (6.9)	
Separated/divorced/widowed	14	4.6	77.3 (7.3)	
Number of people at household				
1	12	4.0	76.7 (8.2)	0.311*
2	109	36.0	77.5 (7.8)	
3	100	33.0	79.0 (6.5)	
4	68	22.4	77.1 (5.6)	
5	11	3.6	80.1 (9.6)	
Monthly family income|| (MW)				
Up to 3.8	16	5.3	77.5 (6.7)	0.516*
3.9 to 5.1	72	23.8	78.9 (7.4)	
5.2 to 6.3	109	36.0	77.3 (7.1)	
More than 6.3	86	28.4	78.0 (6.5)	
Routine medical examinations				
Less than 2 years interval	202	66.7	76.5 (6.0)	< 0.001¶
More than 2 years interval or never	101	33.3	81.0 (8.0)	
Smoking				
Never	255	84.2	78.1 (7.1)	0.678†
Ex-smoker	35	11.6	77.5 (5.5)	
Smoker	13	4.3	76.3 (9.2)	
Alcohol intake				
None or up to once a month	82	27.1	77.4 (7.1)	0.150*
One or more times a week	220	72.6	78.4 (5.5)	
Physical activity				
Yes	177	58.4	77.5 (6.7)	0.189*
No	125	41.3	78.6 (7.6)	
Daily hours of sleep				
6 or more	283	93.4	78.0 (7.1)	0.893*
Less than 6	20	6.6	78.2 (6.8)	
Use of glasses or contact lenses				
No	25	8.3	90.9 (3.8)	< 0.001*
Yes	278	91.7	76.8 (6.0)	

* ANOVA (Levene’s test > 0.05).

† ruskal-Wallistest.

‡ Tukey test: workers with incomplete higher education had a higher average than workers with complete higher education: p = 0.043

§ Tukey test: single workers had a higher average than married workers: p = 0.001

|| Times the minimum wage (MW) at the time of data collection (MW = BRL 788.00).

¶ Mann-Whitney test.

SD = standard deviation.

Clinical visual disturbances found among our 303 participants were myopia (5.11%), hyperopia (27.1%), astigmatism (47.9%), and presbyopia (66.3%). Around 91.7% of them reported wearing corrective lenses (Table l).

Table 2 shows information on continuous variables. The average age of the sample was 46.0 (SD) ± 12.5 years old, varying from 20.0 to 74.0, median 48.0 years old. The mean time working at the current position was 15.8 ± 10.0 years, varying from 0.6 to 44.5, median 15.0 years, and the mean time working at the institution was 18.7 ± 10.2 years, varying from 0.6 to 44.5, median 20.1 years.

**Table 2:** Analysis of correlations between quantitative variables and visual function in computer-based administrative workers of a public hospital, São Paulo, 2015 (n = 303)

Variables	Mean	Median	SD	R*	p-value
Demographic characteristics					
Age (years)	460	48.0	12.5	−0.380	< 0.001
Occupational history characteristics (years)					
Tenure at current position	15.8	15.0	10.0	−0.335	< 0.001
Tenure at the institution	18.7	20.1	10.2	−0.415	< 0.001
Psychosocial factors at work: control-demand model					
Work demands	15.0	16.0	1.4	−0.078	0.176
Control at work	17.1	17.0	2.2	−0.026	0.651
Social support at work	21.2	22.0	2.2	−0.068	0.236
Demand/control ratio	0.89	0.87	0.12	−0.053	0.358
Psychosocial factors at work: effort-reward imbalance model					
Effort	18.4	19.0	2.6	−0.233	< 0.001
Reward	43.4	44.0	3.8	−0.038	0.507
Overcommitment	14.3	15.0	1.7	−0.191	0.001
Effort-reward balance	0.79	0.80	0.15	−0.133	0.021

* Pearson’s correlation coefficient.

SD = standard deviation.

Variables representing the psychosocial environment at work are described in Table 2. The mean score for demand was 15.0 ± 1.4, median 16.0; for control, it was 17.1 ± 2.2, median 17.0, and for social support, it was 21.2 ± 2.2, median 22.0. The mean demand-to-control ratio was 0.89 ± 0.12, median 0.87. The Cronbach’s alpha calculated to assess the reliability of the JSS was over 0.70: demand, α = 0.71; control, α = 0.72) and social support, α = 0.87.

Regarding effort-reward imbalance at work, the average score for effort was 18.4 ± 2.6 (median 19.0); for reward, it was 43.4 ± 3.8 (median 44.0); and for overcommitment, it was 14.3 ± 1.7 (median 15.0). The mean effort-to-reward ratio was 0.79 ±0.1 (median 0.80). The Cronbach’s alpha for ERI exhibited variable values: effort, α = 0.97; reward, α = 0.61; and overcommitment, α = 0.47.

Regarding the participants’ occupational history, Table 3 shows that 62.4% had dual employment contracts and 77.9% worked 40 hours per week. The participants were stratified according to their working conditions: 78.2% of the sample had been using computers at work for 10 years or longer; 80.9% used computers for 5 or more hours a day at work; 98.3% reported they were unable to change postures at work (Table 3).

**Table 3:** Visual function scores of computer-based administrative workers of a public hospital according to occupational characteristics and working conditions, São Paulo, 2015 (n = 303)

Variables	n	%	Mean (SD)	p-value
Employment status				
HCorFZorHC + FZ	17	5.6	82.3 (8.2)	< 0.001*
FFM	97	32.0	80.2 (7.3)	
HC + FFM	189	62.4	76.4 (6.3)	
Weekly working hours at the hospital (hours)				
20	67	22.1	78.7 (7.1)	0.325*
40	236	77.9	77.8 (7.1)	
Computer use at work (years)				
Less than 10	66	21.8	81.8 (7.3)	< 0.001*
10 or more	237	78.2	76.9 (6.6)	
Daily computer use at work (hours)				
Less than 5	58	19.1	78.9 (7.5)	0.294*
5 or more	245	80.9	77.8 (6.9)	
Acoustics at computer location at work				
Optimal	198	65.3	77.3 (6.8)	0.030*
Good/average	105	34.7	79.2 (7.4)	
Lighting at computer location at work				
Optimal	217	71.6	77.9 (7.1)	0.723*
Good/average	86	28.4	78.2 (6.9)	
Temperature at computer location at work				
Optimal	207	68.3	77.8 (6.6)	0.470*
Good/average	96	31.7	78.4 (7.9)	
Possibility to change body postures at work				
Yes	5	1.7	74.8 (3.2)	0.315*
No	298	98.3	78.0 (7.1)	
Chair conditions at work				
Optimal	167	55.1	77.5 (6.5)	0.158*
Good/average	136	44.9	78.6 (7.6)	
Adjustable office chair				
Yes	162	53.5	78.0 (6.8)	0.854*
No	139	45.9	77.9 (7.4)	
Desk conditions at work				
Optimal	168	55.4	77.5 (6.6)	0.219*
Good/average	135	44.6	78.5 (7.6)	
Arms comfortable at work				
Yes	272	89.8	78.1 (7.0)	0.251*
No	30	9.9	76.6 (7.5)	
Workstation layout				
Optimal	187	61.7	77.5 (7.0)	0.092*
Good/average	116	38.3	78.9 (7.1)	
Quality of work tools				
Optimal	186	61.4	77.7 (7.0)	0.317*
Good/average	117	38.6	78.5 (7.1)	

FFM = Fundação Faculdade de Medicina; FZ = Fundação Zerbini; HC = Hospital das Clínicas; SD = standard deviation.

* ANOVA (Levene’s test > 0.05).

Considering visual function, NEI VFQ-25 exhibited satisfactory reliability (α = 0.88): the mean score was 78.0 ±7.1 varying from 50.2 to 99.0, median 77.9. Table 4 shows that the visual function domains with the poorest results were: ocular pain (mean 51.2; SD ± 12.2); difficulty with near vision activities (62.2 ± 15.1); and general health perception (62.8 ± 14.2). The highest scores were found for the color vision (99.0 ± 4.9), dependency (98.5 ± 7.3), and social functioning (91.5 ± 10.9) subscales.

**Table 4:** Scores of visual function scale domains in computer-based administrative workers of a public hospital, São Paulo, 2015

Domains	n	Mean	SD
General health	303	62.8	14.2
Global vision	303	67.1	11.3
Ocular pain	303	51.2	12.2
Near vision activities	303	62.2	15.1
Distance vision activities	303	67.7	12.9
Social functioning	303	91.5	10.9
Mental health	303	81.1	14.3
Role limitations	303	89.9	11.6
Dependency	303	98.5	7.3
Driving	232	75.1	12.9
Color vision	303	99	4.9
Peripheral vision	300	89.4	12.9

SD = standard deviation.

### FACTORS ASSOCIATED WITH VISUAL FUNCTION – UNIVARIATE ANALYSIS

The mean NEI VFQ-25 score of personal characteristics was higher among single employees, those who underwent routine medical examinations, and those who did not wear corrective lenses (Table 1).

Table 2 shows that older employees showed lower NEI VFQ-25 scores. Tenure at both the current position and the institution exhibited statistically significant associations with visual function. Regarding psychosocial factors at work, visual function was inversely correlated with ERI dimensions; the NEI VFQ-25 score was lower when greater effort, greater overcommitment, and greater effort-reward imbalance were observed.

Table 3 describes the results of the univariate analysis of occupational history and working conditions. Employment status exhibited a significant association with visual function; the mean NEI VFQ-25 score was lower among workers with dual employment contracts than among the other employees. The mean NEI VFQ-25 score was lower among employees who reported using computers for 10 years or longer. Acoustic comfort where the computer was located at work was associated with visual function; the mean scores were lower when the acoustic conditions were optimal.

### MULTIPLE LINEAR REGRESSION ANALYSIS (JOINT ANALYSIS OF FACTORS ASSOCIATED WITH VISUAL FUNCTION)

As shown in Table 5, the multiple linear regression analysis indicated that the factors independently associated with visual function were age and effort at work. The NEI VFQ-25 score exhibited a decrease of 0.218 per additional year of age and of 0.656 per additional point in the effort at work score. The adjusted coefficient of determination of the model (r^2^a) was 0.20. Analysis of residuals showed that errors adhered to the normal curve, therefore the model did not show bias.

**Table 5:** Univariate and multiple linear regression analyses of visual function in computer-based administrative workers of a public hospital and independent variables, São Paulo, 2015 (n = 303)

Variables	Univariate				Multiple			
	β	95%CI (β)	p-value	r^2^	β	95%CI(β)	p-value	r^2^a
Age (years)	−0.214	−0.274--0.155	< 0.001	0.14	−0.218	−0.276--0.161	< 0.001	0.20
Tenure at current position (years)	−0.238	−0.313--0.162	< 0.001	0.11	-	-	-	
Tenure at institution (years)	−0.286	−0.357--0.215	< 0.001	0.17	-	-	-	
Employment status	−4.113	-5.695--2.531	< 0.001	0.08	-	-	-	
Computer use at work (years)	−4.851	−6.707--2.995	< 0.001	0.08	-	-	-	
Use of glasses or contact lenses	−14.081	−16.506--11.656	< 0.001	0.30	-	-	-	
Effort	−0.626	−0.922--0.329	< 0.001	0.05	−0.656	−0.928--0383	< 0.001	
Overcommitment	−0.818	−1.293--0.342	0.001	0.03	-	-	-	
Marital status	−3.100	−4.765--1.435	< 0.001	0.04	-	-	-	
Routine medical examinations	4.488	2.871--6.105	0.001	0.09	-	-	-	
Acoustics at computer location at work	1.849	0.183-3.515	0.030	0.01	-	-	-	
Educational level					-	-	-	
Graduate education	−2.107	−4.970--0.756	0.149	0.02	-	-	-	
Incomplete secondary education or less	−2.598	−4.841--0.355	0.023		-	-	-	
Complete higher education	−3.022	−5.278--0.767	0.009		-	-	-	
Workstation layout	1.178	−0.325--2.682	0.092	0.01	-	-	-	
Sex	−1.288	−2.920--0.344	0.122	0.01	-	-	-	
Drinking habits	1.317	−0.478-3.111	0.150	0.00	-	-	-	
Chair conditions at work	1.152	−0.449-2.753	0.158	0.00	-	-	-	
Work demands	−0.381	−0.934-0.172	0.176	0.00	-	-	-	
Physical activity	1.085	−0.538-2.708	0.189	0.00	-	-	-

## DISCUSSION

Our results show a satisfactory quality of visual health among computer-based workers of this public hospital. The factors that remained independently associated with visual function were age and effort at work.

Most of the participants were women, were in the fifth decade of life, and wore corrective lenses. The mean visual function score was similar to those reported by studies with healthy people in Armenia^20^ and people with visual impairment in the United States,^21^ but lower than those in American studies with healthy people^21,22^ and a German population-based study.^23^ The visual function domains with higher scores were color vision, dependency, and social functioning, which was similar to what was observed in a German study.^24^ General health was among the three domains with the lowest scores, just as with participants from Armenia^20^ and Germany.^24^

Some individual, clinical, and work-related variables exhibited statistically significant associations with visual function in our univariate analysis. For example, the sex of participants is not a consensus in the scientific literature – it was associated with visual outcomes in some studies,^7,8^ but did not pose influence in others.^21^ Marital status was not associated with visual function in American studies.^21^ Wearing corrective lenses was associated with visual function in Africa^11^ and Asia.^8^ Screen time (in Spain^7^ and Ethiopia^11^) and time in certain occupations (in Sri Lanka^8^) were associated with visual outcomes such as CVS or asthenopia. However, these variables were eliminated following multiple statistical modeling as a function of the greater effect of age and effort at work in our Brazilian group. The final model showed significance for age and effort, where older age and greater effort corresponded to poorer visual function.

This study showed that, for each additional year of age, there was a statistically significant decrease in mean global NEI VFQ-25 scores. These results are similar to those found in the American^21^ and Armenian^20^ publications. Among our participants, six out of ten had presbyopia. This demonstrated that the workers comprised an aging group in their 4th and 5th decades of life, which was compatible with the expected outcome.^25^ Presbyopia corresponds to the difficulty of clearly distinguishing nearby objects due to the inability to focus the eye to meet the visual demand close by. In our sample group, ocular pain was the most affected domain in visual function, which may be related to exposure to continuous computer-based work associated with presbyopia and other clinical visual disturbances.

Regarding psychosocial factors at work, only effort at work (from the effort-reward imbalance model) was associated with visual function. Effort-reward imbalance and overcommitment were not associated in the multiple model. Similarly to another study that analyzed visual function, ie CVS, the dimensions of the demand-control model were not associated with the ophthalmic outcome (asthenopia).^5^ One study that assessed other psychological factors and visual fatigue among bank employees who used computers at work found that social support, group conflict, poor self-esteem, job dissatisfaction, and skills under-utilization behaved as predictors of visual complaints.^26^

Experimental studies have shown effects of mental overload on visual function in men.^27^ In Norway, young women showed a deficit in visual function when exposed to stress, with effects such as a transient increase in trapezius muscle activity and a more forward leaning posture to try to increase productivity (in relation to reading speed) in people with normal vision.^28^ This impact may be greater in older people with a history of visual impairment, even if corrected. Screen brightness also had a negative influence on visual health, but at the public hospital where this study was conducted, the workstation illuminance assessment (based on institutional documents [PPRA]) showed results of 480 to 500 lux, which are in line with the Brazilian standards.

The following factors were determinants of greater effort at work in the studied population: interruptions at work, working after hours, increase of work demands in the past years, insufficient time for the actual workload, and responsibility at work (data not shown). We believe that the employees’ responses might have been due to changes made at the institution during the period of data collection.

Indeed, by that time (2014-2015) the hospital underwent the implementation and certification of several quality management systems, which might have affected several work processes and changed the hospital organizational charts and procedures. These changes might have further increased a pre-existent excessive workload. In addition, some older employees went into retirement but no new workers were hired in their place. This possibly increased the pressure on the remaining employees to maintain and increase productivity while meeting deadlines. As a result, the participants’ responses reflected this additional degree of effort at work.

A relevant characteristic within this scenario of greater effort at work was an ever-increasing use of computers. Most work requiring computers is associated with considerable mental and especially cognitive demands. Changes in work processes and an increasing use of computers result in greater need for visual efficiency and activation of the nervous system components that coordinate eye movement and accommodation.^16,27,28^

The relationships of personal characteristics of workers, psychosocial risk factors, and environmental factors at the workplace should be accurately evaluated for the purpose of health promotion and prevention of ocular symptoms.^1^ The results of this study thus showed that the effects of computer use on workers’ visual function need to be accurately and regularly assessed and followed up.

Important preventive recommendations in CVS management are: a) regular work evaluations and corrections of environmental conditions^29^ such as screen brightness and contrast adjustment; b) actions to control and reduce negative psychosocial conditions related to work effort^29^; c) promoting eye health education among computer users on preventive strategies that encompass environmental factors and health promotion, including self-assessment^11,29^ and visual rest, such as taking micro breaks, blinking frequency, etc.; d) and the inclusion of the eye exam in periodic examinations of workers.^10,29^ In this last recommendation, the occupational physician must be qualified to perform the measurement of visual acuity. The worker should be referred for a complete ophthalmological examination with an ophthalmologist when: a) presenting visual acuity equal to or less than 20/30 (Snellen’s table) in at least one eye, with or without visual symptoms, or a difference in visual acuity between both eyes of two or more lines; and/or b) presenting strabismus.

In the 21st century, telework is a rapidly growing strategy for increasing productivity. It has become mandatory for the job market, as seen during the COVID-19 pandemic. Computer-based tasks performed at home can be a part of a company policy to improve work-life balance. However, working at home is not without health risks, as it may be performed under sub-optimal work conditions. Recommendations for workers, employers, and public authorities must address advantages and disadvantages of telework.^30^ Suggestions, as well as the support of companies, are welcome to the implementation of home office improvements.

The limitations of the present study include its cross-sectional design, which does not allow the establishment of causal inferences, and the lack of performance of diagnostic tests for dry eye or systemic disorders. Using self-reporting questionnaires could lead to bias, but most of them presented good internal consistency. In regard to its strengths, the present study contributes to the understanding of a subject that is still scarcely investigated; the high rate of participation allows inferring that the results have adequate internal validity, and the robust strategy for statistical analysis minimized bias.

## CONCLUSIONS

The results of the present study showed that despite the aging of the study population, the quality of their visual health was good. They worked at a public hospital, following standards for the promotion and protection of visual health through annual eye examination during a periodic occupational medical consultation. The factors that remained independently associated with visual function were age and effort at work, being that the younger the age and the lower the effort at work, the better the visual function. The results point to the relevance of establishing periodical and preventive health actions, including eye health assessments. Even though its implementation is not a rule, the psychosocial environment at work should be evaluated, especially looking at effort at work, as a strategy for promotion of visual function and prevention of visual disturbances.
